# Incidental radiological discovery of a hydroclayx by a reno-vascular obstruction in a woman: Fraley's syndrome

**DOI:** 10.11604/pamj.2016.24.95.9340

**Published:** 2016-05-27

**Authors:** Amine Slaoui, Ahmed Ibn Attya Andaloussi

**Affiliations:** 1 Urology B Ibn Sina Hospital Rabat, Mohammed V University Rabat, Rabat, Morocco

**Keywords:** Fraley, hydrocalycosis, compression, vascular pedicle

## Image in medicine

A 47-year old patient without significant antecedents consulted for a single episode of hematuria. The urine culture was negative. CT highlighted a hydrocalycosis related to extrinsic compression by a vascular pedicle. It corresponds to Fraley's syndrome. The patient was asymptomatic so the treatment consisted of a survey with regular clinical examinations.

**Figure 1:**
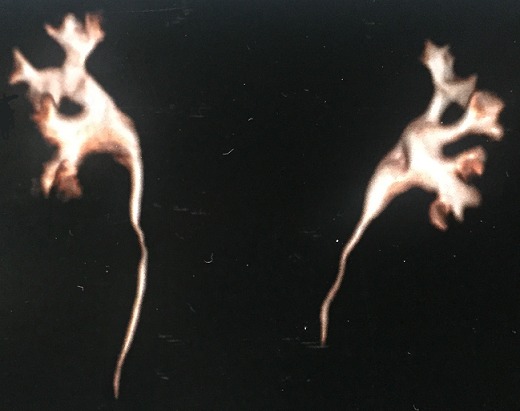
Hydrocalycosis related to extrinsic compression by a vascular pedicle (reconstruction)

